# Rhinovirus infection promotes suppression of sphingosine and enhanced bacterial infection in cystic fibrosis airways

**DOI:** 10.1016/j.jbc.2026.111381

**Published:** 2026-03-19

**Authors:** Gregory C. Wilson, Simone Keitsch, Barbara Wilker, Matthias Soddemann, Markus Kamler, Erich Gulbins

**Affiliations:** 1 Department of Surgery, University of Cincinnati College of Medicine, Cincinnati, Ohio, USA; 2 Institute of Molecular Biology, University Hospital Essen, University of Duisburg-Essen, Essen, Germany; 3 Department of Thoracic and Cardiovascular Surgery, Thoracic Transplantation, University Hospital Essen, University Duisburg-Essen, West German Heart and Vascular Center, Essen, Germany

**Keywords:** *Pseudomonas aeruginosa*, rhinovirus, acid ceramidase, sphingosine, infections

## Abstract

Viral infections often sensitize, *via* unknown molecular mechanisms, the respiratory tract to bacterial infections or induce severe exacerbations in cystic fibrosis (CF). CF, which is caused by inactivation of *Cftr*, affects approximately 80,000 individuals in the United States and Western Europe. Currently, pulmonary complications drive morbidity and mortality for patients with CF, while gastrointestinal problems associated with CF are relatively well-controlled. Here we identify in mouse tracheae and human lung tissue a constitutive upregulation of Stat3 in CFTR/Cftr-deficient cells. This results in activation of interferon regulatory factor 8 (IRF8) and a downregulation of acid ceramidase activity and cellular sphingosine levels. Infection of mouse tracheae with rhinovirus (RV) strains 1B or 2 markedly enhanced these changes in Cftr-deficient cells. Rhinovirus infections also induced Stat3 activation, upregulation of IRF8 expression, down-regulation of acid ceramidase activity and sphingosine levels in airway epithelial cells from wildtype mice, although to a lower level than in CF cells. Inhibition of Stat3 prevented the constitutive and rhinoviral-induced upregulation of IRF8 in CF tracheal epithelial cells and restored acid ceramidase activity and sphingosine levels in these cells to almost normal values. Upregulation of sphingosine in Cftr-deficient epithelial cells by Stat3 inhibition or reconstitution of sphingosine levels by treatment with exogenous sphingosine restored resistance of tracheal epithelial cells to *Pseudomonas aeruginosa* infections. The data suggest that rhinoviral infections facilitate bacterial infections of airway epithelial cells by a cascade consisting of Stat3 activation, IRF8 upregulation, downregulation of acid ceramidase activity, and reduced sphingosine levels.

Cystic fibrosis (CF) is caused by homozygous mutations of the cystic fibrosis transmembrane conductance regulator (human: CFTR, murine: Cftr) (1). With an incidence of one in 2500 births, the disease is the most common autosomal recessive disorder in the EU and the United States, with approximately 80,000 persons affected (1). At present, pulmonary complications remain a significant source of morbidity and mortality for patients with CF, determining overall life expectancy, while the gastrointestinal issues associated with CF are relatively well-controlled. The most significant pulmonary complications are related to chronic inflammation, fibrosis, and recurrent/chronic infections with *Staphylococcus aureus* (*S. aureus*), *Pseudomonas aeruginosa (P. aeruginosa)*, *Burkholderia cepacia*, *Hemophilis influenzae*, and other bacteria. The molecular mechanisms that lead to CF patients’ high susceptibility to infection are not completely known.

Viral and, in particular, rhinoviral infections can contribute to disease progression in CF and often cause pulmonary exacerbations and bacterial pneumonia (2, 3, 4, 5). Rhinovirus is the most common virus detected in the airways of patients with CF (2, 4). Surprisingly, the mechanisms that mediate prolonged viral infections in CF, pulmonary exacerbations of pre-existing bacterial infections, and bacterial pneumonia are poorly characterized. Rhinoviral infections exacerbate the preexisting inflammation seen in CF (2, 4). A study investigating the transcriptome in CF and non-CF epithelial cells in response to rhinovirus infections reported a change in many pathways but did not define pathogenic mechanisms (4). Rhinoviruses seem to impair the function of alveolar macrophages (5), but the effects of rhinoviral infection on epithelial cells are largely unknown.

Several studies have demonstrated an increase of ceramide in ciliated airway epithelial cells and macrophages from both mice and patients with CF using lung tissue, nasal polyps, isolated nasal epithelial cells, alveolar macrophages, and cultured airway epithelial cells from different genetic models of *Cftr*-deficient mice and from (6, 7, 8, 9, 10, 11, 12, 13, 14, 15, 16, 17). Detailed mass spectrometry data of ceramide species in the lungs of patients with CF (10) showed an increase of C16, C18, and C20 ceramides, which might indicate a distinct function of different ceramides. Additionally, unbiased metabolomic studies demonstrated accumulation of ceramide in human CF lungs and also an increase of ceramide in sputum of patients with CF after exacerbation of the disease (16, 17). Guilbault *et al.* demonstrated an increase of dihydro-ceramide and revealed an imbalance of long-chain ceramides (C14 to C20) and very long chain ceramides (C22 and C24) in CF cells (18, 19). This increase in ceramide levels was shown to be important in the pathogenesis of chronic inflammation in the airways of *Cftr*-deficient mice, and also in the induction of susceptibility to pathogenic bacteria such as *S. aureus* and *P. aeruginosa* at least in CF mice (6, 20).

In contrast, sphingosine is markedly decreased in nasal, tracheal and bronchial ciliated epithelial cells of CF mice and in nasal and bronchial epithelial cells from humans, compared to levels in healthy mouse or human airways (21, 22, 23). The imbalance of ceramide and sphingosine in CF cells seems to be caused by a downregulation of the function and expression of the acid ceramidase (15, 20, 23), which converts ceramide into sphingosine.

Treatment of CF mice with inhaled sphingosine restored the ability of airway epithelial cells to kill *P. aeruginosa* and *S. aureus* (21, 23), indicating that it is the lack of sphingosine in CF epithelial cells that results in the high susceptibility of CF mice to bacterial airway infections with *S. aureus* and *P. aeruginosa*.

We, and others, have shown that sphingosine efficiently kills many bacterial species both *in vitro* and *in vivo*, including *P. aeruginosa*, *S. aureus, Staphylococcus epidermidis, Hemophilus influenzae, Escherichia coli, Moraxella catarrhalis*, *B. cepacia*, *Acinetobacter baumanii, Porphyromonas gingivalis*, *and different Mycobacteria species* (20, 21, 22, 23, 24, 25, 26, 27, 28, 29, 30, 31). Sphingosine is abundantly expressed on the luminal surface of human and murine nasal, tracheal and bronchial epithelial cells, while the levels of sphingosine are very low in the corresponding epithelial cells from patients with CF and CF mice (21, 22, 23). Treatment with inhaled sphingosine or acid ceramidase in CF mice eliminates existing and prevented new *P. aeruginosa* and *S. aureus* infections or infections with atypical mycobacteria species, respectively (21, 22, 23, 30, 31). The safety of inhaled sphingosine has been documented in porcine models without adverse effects on epithelial cells of the respiratory tract (29).

We have previously shown that interferon regulatory factor 8 (IRF8) is upregulated in CFTR/Cftr-deficient epithelial cells and controls expression of acid ceramidase activity and thereby sphingosine levels (32). The down-regulation of acid ceramidase expression in CFTR/Cftr epithelial cells of the tracheae and bronchi resulted in a concomitant reduction of sphingosine levels (32). Genetic downregulation of IRF8 corrected the expression levels of acid ceramidase and sphingosine and restored resistance to bacterial infections (32).

It is currently unknown whether a downregulation of the acid ceramidase also plays a role in the susceptibility of patients with CF for viral infections, and in particular, whether the acid ceramidase is involved in the interplay of viral and bacterial pathogens in the airways.

Here, we demonstrate that rhinoviral infections of tracheal epithelial cells result in a Stat3-and IRF8-dependent downregulation of acid ceramidase activity and sphingosine levels in *Cftr-*deficient epithelial cells. Rhinoviral infections induce a down-regulation of acid ceramidase activity and sphingosine in *Cftr*-deficient epithelial cells, driving increased susceptibility to *P. aeruginosa* infection. Thus, prevention of Rhinovirus-induced downregulation or reconstitution of sphingosine levels in the CF airway prevents post-viral *P. aeruginosa* infection.

## Results

**Figure 1:**
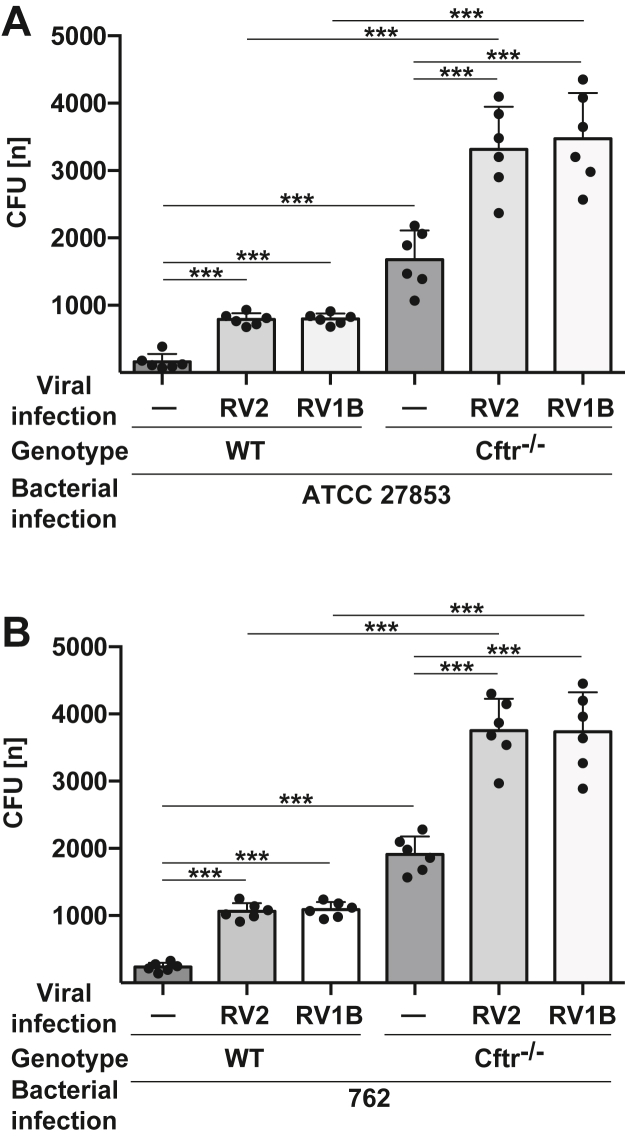
**Rhinoviral infections impair anti-bacterial functions, particularly in CF tracheae.** Tracheae were isolated from wild-type (WT) and CF (Cftr^−/−^) mice and infected with 1 × 10^5^ PFU/ml of rhinovirus RV2 or RV1B for 4 h, washed, and then infected with *P. aeruginosa* ATCC 27853 (*A*) or 762 (*B*) at an MOI of 0.2 (0.2 bacteria per cell) for 60 min. Cells from the tracheae were lysed with saponin, vortexed, centrifuged, pellets were resuspended and aliquots of the samples were cultured on agar plates. *P. aeruginosa* colony-forming units (CFU) were counted after overnight growth. Shown are the mean ± SD of the quantitative analysis of each 6 independent experiments. ∗∗∗*p* < 0.001, ANOVA and *post hoc t* test.

Clinical observations indicate that respiratory tract infections with rhinovirus sensitize CF patients to bacterial respiratory tract infections or exacerbations of existing bacterial pulmonary infections (2, 3, 4, 5). We therefore tested whether infections of wild-type or CF tracheal epithelial cells with Rhinovirus strain 2 (RV2) or rhinovirus strain 1B (RV1B) increase the sensitivity of the epithelial cells to *P. aeruginosa* infections. We used freshly isolated tracheae and infected the epithelial surface of the tracheae. These surfaces contain mostly ciliated epithelial cells but also basal, Clara, goblet, and (rare) neuroepithelial cells. We infected freshly isolated tracheae from wild-type and CF mice for 4 h with RV2 or RV1B, washed the samples and determined the susceptibility to a subsequent infection with *P. aeruginosa* strain ATCC 27853 or the clinical isolate 762. The results revealed a marked increase in infection susceptibility of CF tracheae after a primary rhinoviral infection (Fig. 1, *A* and *B*). Viral infections also increased infection susceptibility of tracheae to *P. aeruginosa* in wild-type mice, although less than in the CF mice.

We have previously shown that deficiency of Cftr results in downregulation of the activity of the lysosomal enzyme acid ceramidase, while the activity of the acid sphingomyelinase, also a lysosomal enzyme, does not seem to be markedly changed (6, 23). This is consistent with an increase of ceramide levels, including some ceramide species seen in CFTR/Cftr-deficient epithelial cells (10, 11, 12, 13, 14, 15, 16, 17, 6, 7, 8, 9). Reduced activity of acid ceramidase also results in a marked downregulation of sphingosine in tracheal and bronchial epithelial cells of Cftr-deficient mice (21, 22, 23) and in lung tissue from human CF patients (23). Sphingosine has been shown to kill many bacterial species, including *P. aeruginosa*, *S. aureus*, and atypical Mycobacteria (24, 25, 26, 27, 28, 29, 30, 31), strains typically associated with CF pneumonia.

**Figure 2:**
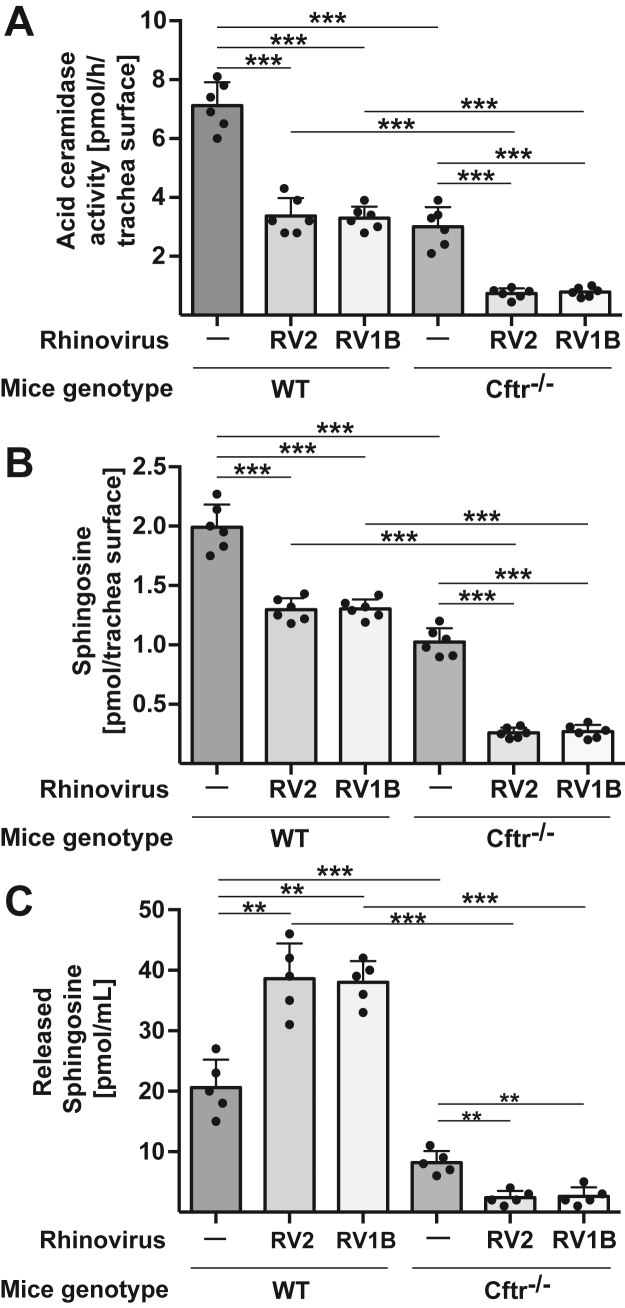
**Rhinoviral infections downregulate acid ceramidase activity and sphingosine levels on the surface of tracheal epithelial cells.** Rhinoviral infection of murine tracheae induced a downregulation of acid ceramidase surface activity and sphingosine surface levels. Changes were most pronounced in the cells from CF mice. Tracheae were isolated from wildtype (WT) and CF (Cftr^−/−^) mice and infected *in vitro* with 1 × 10^5^ PFU/ml of rhinovirus strain RV2 or RV1B for 4 h. *A*, acid ceramidase activity was measured by incubation of the tracheal surface with [^14^C]C_16_ceramide and determination of its consumption *in situ*. *B*, surface sphingosine was determined by incubation of tracheal surface with sphingosine kinase [^32^Pγ]ATP for 15 min. The samples were then extracted and [^32^P]-S1P was determined by thin layer chromatography. Shown are the mean ± SD of the quantitative analysis of each 6 independent experiments; ∗∗∗*p* < 0.001, ANOVA and *post hoc t* test. *C*, to determine the release of sphingosine from tracheae upon infection, isolated tracheae were infected as described above with rhinovirus strains RV2 or RV1B for 4 h, the supernatants collected, microparticles were prepared by centrifugation at 24,600*g* for 30 min and sphingosine concentrations were determined by sphingosine kinase assays. Shown are the mean ± SD of the quantitative analysis of each 5 independent experiments; ∗∗*p* < 0.01, ∗∗∗*p* < 0.001, ANOVA and *post hoc t* test.

To further investigate the molecular mechanisms that mediate the effect of rhinoviral infections on sensitivity/resistance of airway epithelial cells to *P. aeruginosa* infections, we determined acid ceramidase activity and sphingosine concentrations in epithelial cells of wild-type and CF tracheae prior to and after infection with RV2 and RV1B. Surface activity of acid ceramidase was already constitutively lower in tracheae of CF mice compared to wild-type mice (Fig. 2*A*). Infection with RV2 or RV1B reduced the epithelial surface activity of acid ceramidase in both wild-type and Cftr-deficient tracheal cells, but much stronger in Cftr-deficient cells, resulting in very low acid ceramidase activities on the surface of epithelial cells from Cftr-deficient tracheae infected with rhinovirus (Fig. 2*A*).

Likewise, sphingosine levels on the tracheal surface were lower in cells from the Cftr-deficient tracheae compared to wild-type epithelial cells (Fig. 2*B*). Infection of isolated tracheae with RV2 or RV1B also demonstrated reduced surface levels of sphingosine in the tracheal cells of both wild-type and Cftr-deficient mice, with a particularly marked reduction seen in the Cftr-deficient tracheal cells (Fig. 2*B*).

In the studies above, we determined surface sphingosine and showed that both are reduced in epithelial cells but to a much higher degree in the CF epithelial cells upon infection with RV2 or RV1b. We further investigated whether this reduction in cellular sphingosine levels could be related to a release of sphingosine from the epithelial cells into the tracheal lumen. Thus, we tested whether rhinoviral infections induce a release of sphingosine-containing microparticles from epithelial cells into the supernatant. The results show that RV2 and RV1b induced a moderate release of sphingosine from wild-type tracheal epithelial cells (Fig. 2*C*). Tracheal cells from CF mice released much lower amounts of sphingosine-containing microparticles compared to wild-type cells, which were even further reduced in CF tracheae upon infection with RV2 or RV1B (Fig. 2*C*). This excludes the release of sphingosine as the main cause of reduced sphingosine levels in epithelial cells from CF mice with RV2- or RV1B-infection.

**Figure 3:**
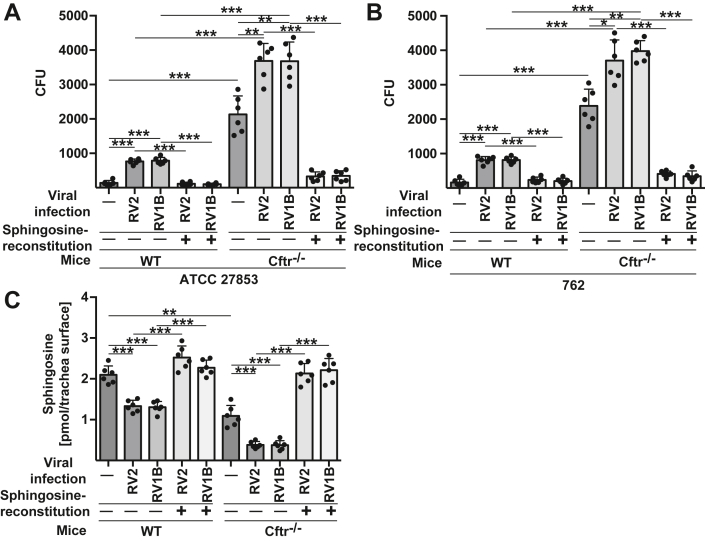
**Restoration of sphingosine in tracheae restores resistance to infection upon viral infections.***A* and *B*, isolated wildtype (WT) or *Cftr*-deficient (Cftr^−/−^) tracheae were infected with RV2 or RV1B for 4 h. Sphingosine was then restored to concentrations comparable to those in uninfected wildtype tracheal epithelial cells by addition of micellar sphingosine (*C*). Controls were left untreated. The tracheae were washed and infected with *P. aeruginosa* at a MOI of 0.2 for 60 min and the CFU of bacteria in the co-cultures was determined. *C*, measurements of sphingosine levels on tracheal surfaces confirm the uptake of sphingosine into tracheal epithelial cells upon incubation with micellar sphingosine. Cells were incubated with 1 μM sphingosine for 15 min, washed and subjected to a surface kinase assay. Samples were then extracted and separated by thin layer chromatography to quantify sphingosine. Shown are the mean ± SD of the quantitative analysis of six independent experiments; ∗*p* < 0.05, ∗∗*p* < 0.01, ∗∗∗*p* < 0.001, ANOVA and *post hoc t* test.

To determine the significance of sphingosine in *P. aeruginosa* infections after rhinoviral infection, we examined the effects of sphingosine treatment on *P. aeruginosa* infection. Isolated Cftr-deficient tracheae were infected with RV2 or RV1B for 4 h, washed and then treated with micellar sphingosine to restore sphingosine concentrations. Controls were left untreated. The tracheae were washed and then infected with *P. aeruginosa,* and bacterial counts were determined. The results show that reconstitution of sphingosine in rhinovirally infected tracheae greatly reduced *P. aeruginosa* infection, particularly in the CF cells (Fig. 3, *A* and *B*). The reconstitution of surface sphingosine levels after treatment with micellar sphingosine was confirmed by *in situ* kinase assays (Fig. 3*C*).

**Figure 4:**
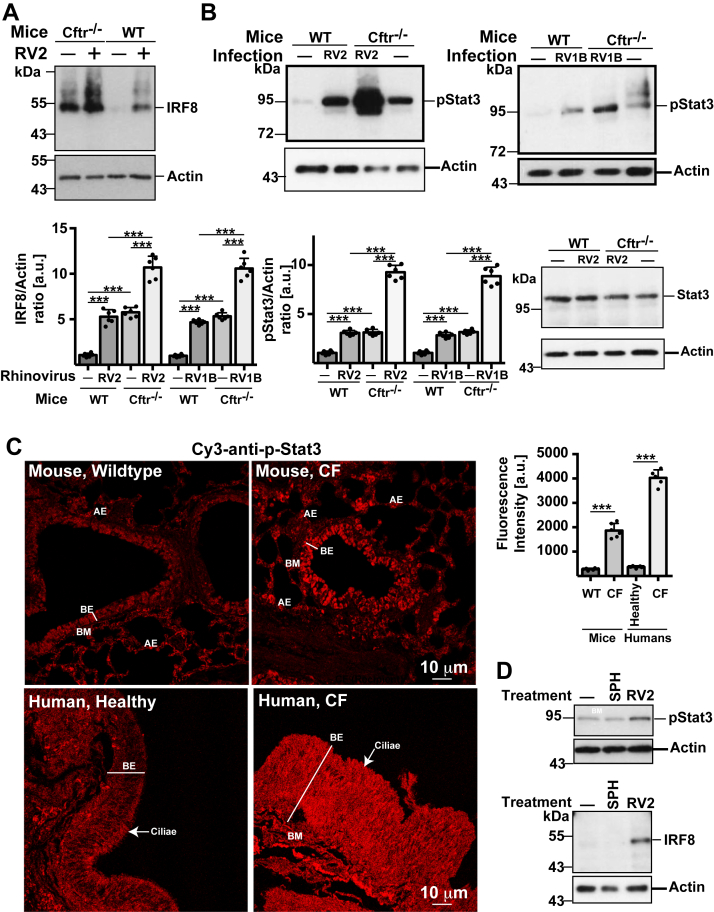
**Rhinoviral infections up-regulate IRF8 and phosphorylation of Stat3.** Rhinoviral infection of murine tracheae induces an increase of IRF8 expression (*A*) and a phosphorylation of Stat3 (p-Stat3) (*B* and *C*). Changes were most pronounced in CF tracheal epithelial cells, which already showed a constitutively increased expression of IRF8 and phosphorylation of Stat3. Tracheae were isolated from wildtype (WT) and CF (Cftr^−/−^) mice and infected with 1 × 10^5^ PFU/ml of rhinovirus strain RV2 or RV1B for 4 h. Samples were lysed and IRF8 or p-Stat3 were determined by western blotting (*A* and *B*). Controls (*lower right panel* in *B*) indicate that infection of tracheae with RV2 did not change Stat3 expression in wildtype or CF tracheae (*B*). Phosphorylation of Stat3 in wildtype and CF mice as well as healthy and CF humans was also determined by immunofluorescence staining of histological lung sections with Cy3-coupled anti-p-Stat3 antibodies. Sections are labeled for the bronchial epithelial cell layer (BE), alveolar epithelial cells (AE) and the basal membrane (BM). Sections in the human samples are from large bronchi and contain mostly ciliated epithelial cells (*C*). Treatment of isolated tracheae with sphingosine (SPH) did not change phosphorylation of Stat3 or expression of IRF8. (*D*) Infection with RV2 served as positive control (*D*). Shown are a representative examples and the mean ± SD of the quantitative analysis of each 6 (*A*, *B*, and *D*) or 5 (*C*) independent experiments or samples, respectively. Actin levels served to normalize samples. The quantitative data in panel *C* are from different human lungs and mice, respectively. ∗∗∗*p* < 0.001, ANOVA and *post hoc t* test. Scale bar: 10 μm.

Next, we further explored the molecular mechanisms that mediate the sensitivity of respiratory epithelial cells to bacterial infections after rhinovirus infections. We have previously shown that deficiency of Cftr results in an upregulation of IRF8, which mediates the downregulation of acid ceramidase activity (32). Therefore, we tested whether infection of tracheal epithelial cells with RV2 or RV1B alters the expression levels of IRF8. The results reveal a strong increase in IRF8 expression in Cftr-deficient tracheal cells upon infection with RV2 and RV1b (Fig. 4*A*). Wild-type epithelial cells also responded to RV2 and RV1B infection with an upregulation of IRF8, but to a lesser extent than Cftr-deficient cells (Fig. 4*A*).

Next, we aimed to identify mechanisms how rhinovirus induces IRF8. To this end, we determined the activity of the transcriptional regulator, Stat3, prior and after RV2 application to isolated tracheae. The results show a marked upregulation of Stat3 phosphorylation in wildtype tracheae after infection with RV2. Stat3 phosphorylation was already constitutively increased in Cftr-deficient tracheal epithelial cells (Fig. 4*B*), and markedly increased after infection with RV2 and RV1B (Fig. 4*B*); mirroring the pattern observed for IRF8 expression. Controls show that the expression of Stat3 did not change in tracheal epithelial cells after infection of wildtype or CF tracheae with RV2 (Fig. 4*B*). Stat3-phosphorylation was also increased in human lungs from individuals with CF (explanted tissue) compared to healthy lungs (donor tissue) (Fig. 4*C*). Sphingosine treatment itself did not change phosphorylation of Stat3 or expression of IRF8 in mouse tracheae (Fig. 4*D*).

To test the significance of Stat3 in IRF8 upregulation and acid ceramidase and sphingosine downregulation, we inhibited Stat3 with a cell-permeable inhibitory peptide, consisting of the sequence PpYLKTK-mts (33). This peptide inhibits the recruitment of STAT3 to the activating Jak2 kinase and thereby prevents phosphorylation of STAT3 at Y705 and its activation (33).

**Figure 5:**
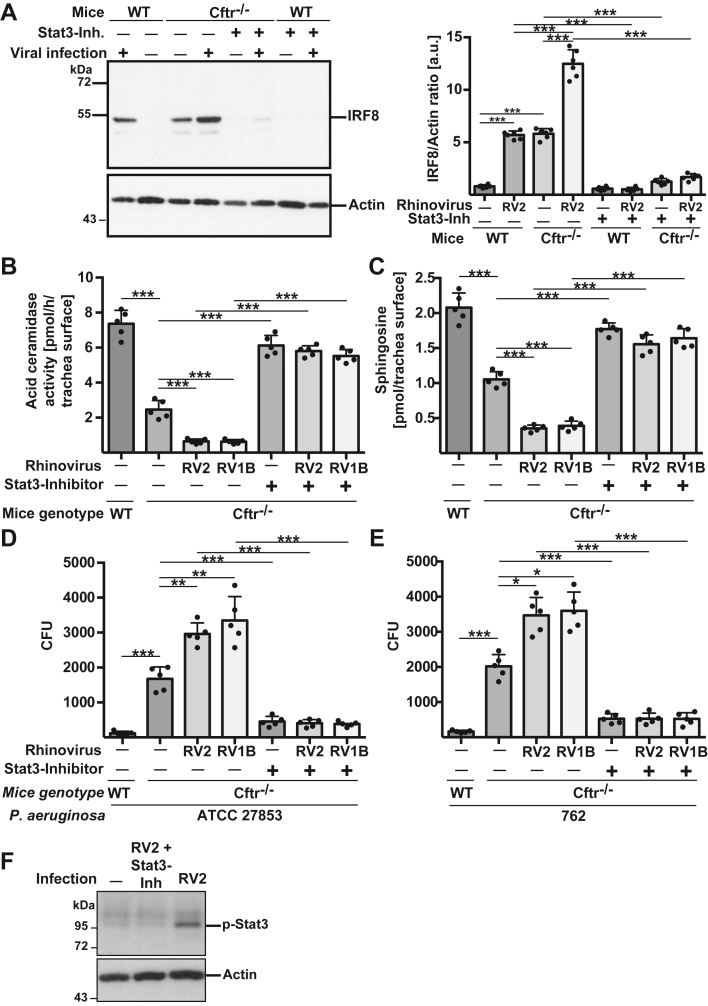
**Cftr deficiency regulates IRF8, acid ceramidase activity, sphingosine levels, and infection-susceptibility *via* Stat3 activity.** Rhinovirally induced increased expression of IRF8 (*A*), downregulation of acid ceramidase surface activity (*B*), and surface levels of sphingosine (*C*) in wild-type and CF tracheal cells are normalized by inhibition of Stat3 activity using a cell-permeable Stat3 inhibitory peptide. Stat3 inhibition also normalized the constitutive increase of IRF8 expression and the constitutive decrease of acid ceramidase surface activity and sphingosine surface levels in CF cells. IRF8 was determined by Western blotting of tracheal lysates (*A*), acid ceramidase activity on the surface of the tracheae by an enzymatic *in situ* assay using [^14^C]C_16_ceramide as substrate (*B*), sphingosine on the tracheal surface by an *in situ* kinase assay (*C*). Normalization of these alterations in CF cells or after rhinoviral infection also normalized the susceptibility to *P. aeruginosa* strain ATCC 27853 (*D*) and 762 (*E*) of wild-type and CF tracheal cells after primary rhinoviral infection. Wild-type or CF tracheae were infected with rhinovirus strains RV2 or RV1B, washed and infected with *P. aeruginosa* strains ATCC 27853 or 762 at a MOI of 0.2 for 60 min. The CFU of bacteria in the co-cultures was determined. *F*, controls show that the STAT3-inhibitory peptide prevented Stat3 phosphorylation at Y705. Tracheae from wildtype mice were infected with RV2 in the presence or absence of the inhibitory peptide or left untreated. Lysates were obtained as above and blotted with anti-p-Stat3 antibodies. Blots are representative of four independent experiments with similar results. Shown are a representative example and the mean ± SD of the quantitative analysis of each 6 independent experiments; ∗*p* < 0.05, ∗∗∗*p* < 0.001, ANOVA and *post hoc t* test.

Pharmacological inhibition of Stat3 activity prevented the constitutive upregulation of IRF8 in *Cftr*-deficient tracheal epithelial cells and abolished the upregulation of IRF8 in wildtype and Cftr-deficient tracheal epithelial cells after rhinoviral infection (Fig. 5*A*). In addition, pharmacological inhibition of Stat-3 in Cftr-deficient tracheal epithelial cells almost normalized surface activity levels of acid ceramidase (Fig. 5*B*) and surface epithelial sphingosine levels (Fig. 5*C*).

Most importantly, inhibition of Stat3 restored the resistance of virally-infected or non-infected *Cftr*-deficient tracheal epithelial cells to *P. aeruginosa* ATCC 27853 (Fig. 5*D*) or 762 (Fig. 5*E*) infections to sensitivity levels observed in wildtype tracheal epithelial cells (Fig. 5, *D* and *E*).

Since the level of Stat3 phosphorylation at Y705 is not only a good measurement for the activity of Stat3, but also for the inhibition of Stat3 by this peptide, we performed Western blots with anti-phospho-Stat3 after viral infection in the presence or absence of the inhibitory peptide. The results reveal that the inhibitory peptide prevented phosphorylation of Stat3 at tyrosine 705, indicating inhibition of Stat3 (Fig. 5*F*).

## Discussion

Rhinoviral infections often sensitize patients with CF to subsequent bacterial infections or trigger severe exacerbation of a preexisting chronic infection with *P. aeruginosa* (2, 3, 4, 5). Rhinoviral infections also sensitize healthy individuals to subsequent bacterial infections. While the clinical observation is well known, the underlying molecular mechanisms are poorly defined. In the present study, we demonstrate that infection of mouse *ex vivo* tracheal epithelial cells with different rhinovirus strains results in activation of Stat3 and a down-regulation of IRF8. While these changes are also observed in epithelial cells from wild-type mice, they are much more pronounced in tracheae isolated from Cftr-deficient mice. IRF8 mediated downregulation of acid ceramidase activity (32), resulting in a marked reduction of sphingosine levels in the tracheal epithelial cells upon rhinoviral infection, most pronounced in Cftr-deficient mice.

Sphingosine has been shown by several studies to kill a variety of bacterial pathogens, including *P. aeruginosa*, *S. aureus*, or atypical Mycobacteria, which are the most important pathogens in CF lung infections (1, 34, 35). We have shown that sphingosine binds to cardiolipin in bacterial membranes, induces a degradation of cardiolipin, and induces severe morphological alterations of the bacterial membranes, leading to rapid death of the bacteria (28, 36, 37). This mechanism also explains why sphingosine acts against many bacteria, while mammalian cells that do not contain cardiolipin in the plasma membrane are relatively resistant to sphingosine. Mammalian cells express cardiolipin in the inner mitochondrial membrane, but also express sphingosine-kinase two in mitochondria, which may protect mammalian cells from exogenous or cell surface sphingosine.

Our data indicate that rhinoviral infections increase the down-regulation of acid ceramidase activity and cellular sphingosine levels in CF epithelial cells by phosphorylation and activation of Stat3. Rhinoviral infections of healthy tracheal cells also induce an activation of Stat3, which is similar to that constitutively present in Cftr-deficient cells. Infection of Cftr deficient cells with Rhinovirus adds to the already existing constitutive activation of Stat3 in CF epithelial cells, resulting in a very strong phosphorylation/activation of Stat3. The significance of Stat3 activation for the down-regulation of acid ceramidase activity and sphingosine levels is indicated by the experiments demonstrating that inhibition of Stat3 normalizes acid ceramidase activity and sphingosine levels in Cftr-deficient cells prior to and even after rhinoviral infections.

Our data also indicate that Stat3 mediates its effects on acid ceramidase activity *via* an up-regulation of IRF8. The effect of Stat3 on IRF8 is again markedly up-regulated upon rhinoviral infection. We have previously shown that IRF8 is constitutively up-regulated in human and mouse Cftr-deficient epithelial cells and that the upregulation of IRF8 results in the downregulation of acid ceramidase activity (32). Here, we demonstrate that inhibition of Stat3 in Cftr-deficient cells prevents the constitutive and virally induced upregulation of IRF8. These data suggest that Stat3 transcriptionally regulates the expression of IRF8 in CF epithelial cells, a mechanism that is enhanced upon viral infection, leading to down-regulation of acid ceramidase activity.

In principle, we observe similar findings in wildtype tracheal cells upon rhinoviral infection, but to a lesser degree than in Cftr-deficient cells. This might explain why rhinoviral infections sensitize both healthy individuals and those with CF to subsequent bacterial infections, but certainly to a higher degree in individuals with CF.

Our data are consistent with a previous study in CML cells that showed a marked upregulation of IRF8 and a downregulation of acid ceramidase in these cells (38). The data are also consistent with transcriptome data from patients with mild CF showing an upregulation of IRF1, IRF2, IRF8, and STAT2 transcription factor activity (39).

At present it is unknown how rhinoviral infections regulate Stat3 and IRF8 in the context of Cftr deficiency. It might be possible that Stat3 interacts with RIG-I or other proteins that detect the viral RNA and thereby activate Stat3 to trigger an inflammatory response. The up-regulation of IRF8 would then be part of the inflammatory response and function together, for instance, with activation of the inflammasome. It is also unknown how Cftr deficiency triggers Stat3 activation. CF is characterized by increased susceptibility to bacterial infections, but also by a chronic inflammation. Cftr deficiency has been shown to induce increased ceramide levels in epithelial cells (6). Such an upregulation of ceramide may also occur in lysosomes altering the biophysical properties of lysosomal membranes. The mTorC1 complex can detect changes in lysosomal membranes (40), and it might be possible that alterations of mTorC1 activity in CF cells mediate Stat3 stimulation. This could be mediated by a direct phosphorylation of Stat3 by the activated mTorC1 or indirectly by a mTorC1-mediated inhibition of phosphatases, which then allows Stat3 phosphorylation and activation. Rhinoviral particles mature in late endosomes and early lysosomes, and the RNA is released from the vesicles into the cytoplasm. The membrane changes associated with this release might synergize with the alterations induced by Cftr deficiency and trigger Stat3 activity. This scenario would also explain the additive effects of Cftr deficiency and viral infections but requires extensive studies beyond the present manuscript.

In summary, our data indicate that rhinoviral infections strongly promote the constitutive phosphorylation/activation of Stat3 in Cftr-deficient cells resulting in IRF8 upregulation, down-regulation of acid ceramidase activity and decreased cellular sphingosine levels. The marked reduction of sphingosine in Cftr-deficient cells mediates at least some parts of the infection susceptibility of these cells to *P. aeruginosa* as indicated in our re-constitution experiments.

## Experimental procedures

### Ethic permissions

Studies on human cells and tissues, respectively, were approved by the local ethics committee University Hospital Essen under the number 17-7326-BO. All experiments followed the Declaration of Helsinki principles.

### Human lung sections

Tissue sections were obtained from explanted CF or remaining donor lung tissues. Tissue biopsies were fixed in 4% PFA for 48 h and then embedded in paraffin, sectioned, dewaxed, and stained as described below.

### Mice

B6.129P2(CF/3)-*Cftr*^TgH(neoim)Hgu^ con-genic mice (named CF^MHH^) were generated by inbreeding the original *Cftr*TgH^(neoim)^Hgu mutant mouse strain, which was obtained by insertional mutagenesis in exon 10 of the Cftr gene (41, 42). This congenic *CF*^*MHH*^ strain was backcrossed for more than 20 generations into the B6 background. These mice express low levels of Cftr due to wild-type exon skipping and aberrant splicing (41, 42). Because these mice still express low levels of Cftr, they can be fed a standard mouse diet. Previous studies demonstrated that these mice display some pulmonary pathologies typical of CF, for instance, focal accumulation of mucus (42), increased infection susceptibility, accumulation of peri-bronchial neutrophils and macrophages, as well as increased concentrations of IL-1 β in the lung (6). Syngeneic wild-type B6 littermates were used as controls.

We have previously demonstrated the typical changes of CF, ceramide accumulation, and decreased sphingosine levels that develops over time in these mice (6). All mice used were at least 16 weeks of age and weighed between 25 and 35 g. Both female and male mice were used in these studies. There were no observed differences in ceramide, sphingosine or infection susceptibility between female and male mice. Mice were divided into cages of equal size (usually 3–4 mice/cage) by animal unit technical staff with no involvement in study design. Cages were randomly assigned to an experimental group.

Mice were housed and bred within isolated-ventilated cages in the mouse facility of the University Hospital Essen, or of the University of Cincinnati. They were tested for a panel of common murine pathogens every 6 months according to the 2002 recommendations of the Federation of European Laboratory Animal Science Associations. The mice were always free of any pathogens.

Mice were sacrificed by cervical dislocation prior to removal of the trachea. No experiments on living mice were performed. Organ (trachea) removal was approved by the local IACUC, Cincinnati, USA, and the Internal Review Board of the Animal Facility, University Hospital Essen, Germany.

### Mouse tracheae

Syngeneic wild-type or CF mice were sacrificed, the tracheae were immediately removed, washed once in HEPES/Saline (H/S; 132 mM NaCl, 20 mM HEPES [pH 7.4], 5 mM KCl, 1 mM CaCl_2_, 0.7 mM MgCl_2_, 0.8 mM MgSO_4_), carefully opened in the longitudinal direction to expose the surface and infected or left uninfected in H/S for 4 h. Tracheae were infected with 10^5^ PFU of RV1B or RV2 for 4 h. If indicated, tracheae were incubated during the infection with 1 μM of the cell-permeable Stat3 inhibitory peptide (Merck # 573096) (33). The tracheae were washed to terminate the experiment and used for western blots, measurement of acid ceramidase activity or sphingosine levels or infected with *P. aeruginosa* strains ATCC 27853 or 762 as described below. *P. aeruginosa* infection of the trachea was performed for 60 min after the primary rhinoviral infection or after treatment with sphingosine.

In the sphingosine reconstitution experiments, the tracheae were infected with rhinovirus for 4 h, washed and incubated with 1 μM sphingosine for 15 min. Sphingosine was suspended as a 2.5 mM stock solution in a 0.9% NaCl solution. Before its use, we sonicated the sphingosine stock in a bath-sonicator for 10 min to promote the formation of micelles, and we then diluted the sphingosine stock directly to 1 μM in H/S. The same volume of 0.9% NaCl in H/S as used was added to all control samples. The tracheae were washed after the 15 min incubation period and used for measurement of sphingosine levels or were infected with *P. aeruginosa* strains ATCC 27853 or 762 as described below. *P. aeruginosa* infection of the tracheae was performed for 60 min after the primary rhinoviral infection or treatment with sphingosine.

### Western blots

Tracheae from wildtype or CF mice were removed, washed twice in H/S and infected with 10^5^ PFU RV2 or RV1B in H/S for 4 h. The Stat3 inhibitory peptide (1 μM) was added together with the viruses and maintained during the 4 h incubation period. Samples were then washed twice in H/S and lysed in 25 mM HEPES, 3% NP40, 0.1% Triton X-100, 10 mM EDTA, 10 mM sodium pyrophosphate, 10 mM sodium fluoride, 125 mM NaCl, 20 mM sodium-orthovanadate and 10 μg/ml aprotinin/leupeptin. Lysis was allowed to complete for 5 min at 4 °C, samples were then centrifuged at 14,000 rpm for 5 min at 4 °C and the supernatants were added to 5x SDS-Laemmli buffer. Samples were incubated for 5 min at 95 °C, and proteins were separated by 8.5% or 10% sodium dodecyl sulfate polyacrylamide gel electrophoresis (SDS-PAGE) for the analysis of IRF8, phosphorylation of Stat3, expression of Stat3 or actin expression, respectively. The gels were blotted onto nitrocellulose membranes overnight, blocked in Starting Block Tris-buffered saline (TBS) blocking buffer (ThermoFisherScientific, #37542) for 60 min, washed and incubated for 60 min with anti-IRF8 (Cell Signaling Clone D20D8, #5628), anti-phospho-Stat3 (Tyr705) (Cell Signaling, #9131) antibodies or anti-Stat3 antibodies (Cell Signaling, #9139). All antibodies were diluted 1:1000-fold in Starting Block (TBS) blocking buffer. Blots were washed 5-times in TBS/0.05% Tween and incubated for 60 min with alkaline phosphatase (AP)-coupled anti-rabbit-IgG (1:50,000, Antibodies.com, #294929), washed again 5-times in TBS/0.05% Tween, washed twice in alkaline wash buffer, and developed with the CDP-STAR NitroBlockII Enhancer system (PerkinElmer). Aliquots were separated on 8.5% SDS-PAGE and blotted and incubated for 60 min with HRP-coupled anti-actin antibodies (1:200,000, Santa Cruz Inc., #sc47778) antibodies at room temperature, washed and developed. These samples served as loading controls and to normalize Western blot intensities.

### Acid ceramidase surface activity

Tracheae were opened and infected with rhinovirus RV1B or RV2 or RV14, respectively, for 4 h in the presence or absence of the Stat3 inhibitor as described above, washed twice in 150 mM sodium acetate (pH 5.0) and 5 μl of the reaction buffer consisting of 1 μCi [^14^C_16_]ceramide (55 mCi/mmol, #ARC-0831, ARC), 150 mM sodium acetate and 0.05% octylglucopyranoside was carefully added onto the luminal surface of the trachea. Prior to the assay, the stock [^14^C_16_]ceramide was dried, resuspended in 0.05% octylglucopyranoside in 150 mM sodium acetate (pH 5.0), and bath sonicated for 10 min. Samples were incubated in a humidified chamber for 30 min at 37 °C. To terminate the reaction, tracheae were transferred into 200 μl H_2_O_2_ and the samples were extracted in CHCl_3_:CH_3_OH:HCl (100:100:1, v/v/v). The lower phase was dried, and samples were resuspended in CHCl_3_:CH_3_OH (1:1, v/v), separated by TLC with CHCl_3_:CH_3_OH:NH_4_OH (90:20:0.5, v/v/v), and analyzed with a Fuji Imager.

### Surface sphingosine measurements

After infection and/or treatment, the tracheae were washed twice in kinase buffer consisting of 50 mM HEPES (pH 7.4), 250 mM NaCl, and 30 mM MgCl_2_. Then 5 μl of the kinase buffer containing 1 μU sphingosine kinase (R&D Systems), 1 μM adenosine triphosphate (ATP) and 10 μCi [^32^P]γATP were added to the surface of the trachea and incubated at 37 °C for 15 min. The kinase reaction was terminated by placing the trachea in 100 μl H_2_O, followed by the addition of 20 μl 1N HCl, 800 μl CHCl_3_/CH_3_OH/1N HCl (100:200:1, v/v/v), each 240 μl CHCl_3_ and 2 M KCl. Samples were vortexed, centrifuged, the lower phase was collected, dried, dissolved in 20 μl of CHCl_3_:CH_3_OH (1:1, v/v) and separated on Silica G60 thin layer chromatography (TLC) plates using CHCl_3_/CH_3_OH/acetic acid/H_2_O (90:90:15:5, v/v/v/v) for development. The TLC plates were analyzed using a Fuji phosphoimager.

### Release of sphingosine from tracheal epithelial cells

Tracheae from wild-type CF mice were infected with RV2 or RV1B for 4 h. The supernatants were collected and centrifuged at 24,600*g* for 30 min to obtain microparticles. The microparticles were then extracted, and sphingosine was determined as described above.

### Immunohistochemistry of human and mouse lungs

Staining was performed as previously reported (6, 23). For immunohistochemical evaluation of murine tracheae, mice were sacrificed by cervical dislocation, the trachea removed and fixed for 36 h with 4% PBS-buffered paraformaldehyde (PFA; Roth, #0335.3). Freshly obtained human lung tissue samples were fixed in 4% PBS-buffered paraformaldehyde for 48 h. Samples were then serially dehydrated with an Ethanol to Xylol gradient, embedded in paraffin, sectioned at 7 μm, dewaxed, and rehydrated. The samples were then incubated with pepsin (Digest All; Invitrogen, #003009) for 30 min at 37 °C to retrieve antigens, washed in PBS 5-times and unspecific antibody binding sites were blocked for 15 min at room temperature with PBS and 5% fetal calf serum (FCS). The samples were stained with rabbit anti-phospho-Stat3 antibodies (Cell Signaling, #9131) in H/S plus 1% FCS at room temperature for 45 min. Samples were washed three times with PBS plus 0.05% Tween 20 and once with PBS. Samples were then incubated for 45 min with Cy3-coupled anti-rabbit F(ab)_2_ fragments (1:500; Jackson Immunoresearch, #711-166-152) in H/S plus 1% FCS, washed three times with PBS plus 0.05% Tween 20 and once with PBS and finally embedded in Mowiol. Samples were evaluated by confocal microscopy using a Leica TCS-SL confocal microscope equipped with a 40× lens, and images were analyzed with Leica LCS software version 2.61 (Leica Microsystems). All comparative samples were measured at identical settings.

All immunostainings were controlled with isotype control rabbit IgG, which showed no or very weak staining. We also included controls with secondary Cy3-coupled antibodies only. These controls confirmed the specificity of the staining and revealed no significant background staining.

### *P. aeruginosa* infections

*P. aeruginosa* strain American Type Culture Collection (ATCC) 27853, a laboratory strain, or the clinical isolate named 762 (43) were grown overnight on tryptic soy agar (TSA; Becton Dickinson Biosciences, Heidelberg, Germany), removed from the plate, resuspended in tryptic soy broth (TSB, Becton Dickinson Biosciences) at an optical density (OD) at 550 nm of 0.2 to 0.25 and grown for 1 h at 37 °C with 125 rpm shaking. The bacteria reach the early logarithmic phase in this time, allowing reproducible conditions for infection. Bacteria were centrifuged at 1710*g* for 10 min, washed once in sterile H/S, resuspended in H/S, and the tracheae were infected for 60 min at a ratio of 0.2 bacteria: one cell, *i.e.* a multiplicity of infection (MOI) of 0.2. The approximate cell number in the surface layer was determined by multiplying the length and width of the trachea by that of an epithelial cell (approximately 10 μM). After the infection, samples were treated with 5 mg/ml saponin, which lyses the mammalian cells, but does not affect bacteria, to release any intracellular bacteria. Samples were then washed twice in sterile H/S by 10 min centrifugation at 3000 rpm x g, the pellets were resuspended in sterile H/S, plated on TSB plates, and colonies were counted after o/n growth.

### Quantification and statistical analysis

All data are expressed as arithmetic means ± SD. For the comparison of continuous variables from independent groups, we used one-way ANOVA followed by *post hoc* Student´s *t*-tests for all pairwise comparisons and the Bonferroni correction for multiple testing. The *p* values for the pairwise comparisons were calculated after Bonferroni correction. All values were normally distributed. Statistical significance was set at the level of *p* ≤ 0.05 (two-tailed). Sample size planning for the continuous variable was based on two-sided Wilcoxon-Mann-Whitney tests (free software: G∗Power Version 3.1.7, University of Duesseldorf, Germany). Blots and fluorescence staining were quantified using MultiGauge V3.0. Investigators were blinded to the identity of the samples in all microscopy experiments.

## Data availability

All data are available on request from the authors. Authors confirm that all data are included in the manuscript.

## Conflict of interests

The authors declare that they have no conflicts of interest with the contents of this article.
